# 

**Published:** 1854-03

**Authors:** 


### BOOKS RECEIVED.

We have received a great many books of late which we can only acknowledge in the present number. We shall speak more particularly of them hereafter.

We are indebted to Dr. Laroche, of Philadelphia, for an interesting monograph on Pneumonia and Autumnal fevers, in their relation to Malaria.

Dr. Bennet Dowler has sent us his "Tabuleau of the Yellow Fever, of New Orleans, in 1853," which is distinguished for research, as are all the productions of this laborious physician.

Our thanks are due to Dr. Fenner for an able monograph on the same subject. We hope to find room for an abstract of both these works in our next number.

Prof. Flint has favored us with Reports on Dyssentery and Chronic Pleurisy. These reports are based on the analysis of numerous cases, and will be found to embrace many valuable facts, and to be singularly free from vague generalizations.

Besides these, we have also received the profound treatise of Mr. LAWRENCE on Diseases of the Eye, edited by Dr. ISAAC HAYS, a standard work on this important subject; the second volume of PEREIRA's encyclopædian treatise on Materia Medica; a treatise by M. A. VIDAL (*De Cassis*) on Venereal Diseases, translated and edited by Dr. GEORGE C. BLACKMAN, of New York, who has made valuable additions to the original; a Practical Treatise on Inflammation of the Uterus, by Dr. JAMES HENRY BENNET, who has become so eminent for his attainments in this speciality; the Lectures on Surgical Pathology, by Mr. PAGET; a Manual of Obstetrics by Dr. T. F. COCK, of New York; a new edition of ELLIS' popular Medical Formulary; a new edition of BUDD's admirable work on the Liver; and a treatise on Diseases of the Ear, by Mr. WILDE, Fellow of the Royal College of Surgeons, of Ireland, &c.

To these, we have to add the Seventh Annual Report of the Board of Regents of the Smithsonian Institution, for the year 1852; and the Annual Reports of the Pennsylvania and Indiana Hospitals for the Insane. Just as this number was going to press, we had the pleasure to receive from our friend, Dr. W. L. SUTTON, the First Annual Report on the Births, Marriages and Deaths, in Kentucky, from January 1, 1852, to December 31st, 1853, digested and prepared by that indufatigable physician. The mass of statistics herein presented by Dr. SUTTON is full of interest and instruction. We shall hasten to avail ourselves of it.

